# Single Low-Density Lipoprotein Apheresis Does Not Improve Vascular Endothelial Function in Chronically Treated Hypercholesterolemic Patients

**DOI:** 10.1155/2016/4613202

**Published:** 2016-02-22

**Authors:** Kevin D. Ballard, Eunice Mah, Yi Guo, Richard S. Bruno, Beth A. Taylor, Jo Ellen Beam, Donna M. Polk, Paul D. Thompson

**Affiliations:** ^1^Department of Kinesiology and Health, Miami University, Oxford, OH 45056, USA; ^2^Division of Cardiology, Hartford Hospital, Hartford, CT 06102, USA; ^3^Human Nutrition Program, Department of Human Sciences, The Ohio State University, Columbus, OH 43210, USA; ^4^Department of Kinesiology, University of Connecticut, Storrs, CT 06269, USA; ^5^Division of Cardiology, Brigham and Women's Hospital, Boston, MA 02115, USA

## Abstract

*Objective*. To investigate vascular endothelial function (VEF) responses to a single low-density lipoprotein (LDL) apheresis session in hypercholesterolemic patients undergoing chronic treatment.* Methods*. We measured brachial artery flow-mediated dilation (FMD), plasma lipids, vitamin E (*α*- and *γ*-tocopherol), markers of oxidative/nitrative stress (malondialdehyde (MDA) and nitro-*γ*-tocopherol (NGT)), and regulators of NO metabolism (arginine (ARG) and asymmetric dimethylarginine (ADMA)) prior to (Pre) and immediately following (Post) LDL apheresis and at 1, 3, 7, and 14 d Post in 5 hypercholesterolemic patients (52 ± 11 y).* Results*. Relative to Pre, total cholesterol (7.8 ± 1.5 mmol/L) and LDL-cholesterol (6.2 ± 1.2 mmol/L) were 61% and 70% lower (*P* < 0.01), respectively, at Post and returned to Pre levels at 14 d. Brachial FMD responses (6.9 ± 3.6%) and plasma MDA, ARG, and ADMA concentrations were unaffected by LDL apheresis. Plasma *α*-tocopherol, *γ*-tocopherol, and NGT concentrations were 52–69% lower at Post (*P* < 0.01), and *α*-tocopherol remained 36% lower at 1 d whereas NGT remained 41% lower at d 3.* Conclusions*. Acute cholesterol reduction by LDL apheresis does not alter VEF, oxidative stress, or NO homeostasis in patients treated chronically for hypercholesterolemia.

## 1. Introduction

Lowering of plasma low-density lipoprotein-cholesterol (LDL-C) concentrations by chronic LDL apheresis therapy retards coronary atherosclerotic plaque progression [1] and reduces the risk of future coronary events in patients with familial hypercholesterolemia (FH) [2] who are refractory or intolerant to other lipid-lowering therapies. Indeed, plasma LDL-C concentrations decrease by 70% immediately following a single LDL apheresis treatment session [3], followed by a return to pretreatment levels within 2 weeks, suggesting that chronic therapy is necessary to reduce cardiovascular disease (CVD) risk in FH patients.

A single LDL apheresis session improves vascular endothelial function (VEF) measured by acetylcholine-induced forearm vasodilation in FH patients undergoing chronic treatment [4]. This effect occurs, at least in part, by decreasing oxidative stress responses [4] that otherwise inhibit nitric oxide (NO) synthesis, an antiatherogenic mediator [5]. The duration of these benefits in chronically treated patients following a single LDL apheresis session has not been investigated. Brachial artery flow-mediated dilation (FMD), a noninvasive measure of VEF that is predictive of future CVD events [6], reflects NO bioavailability [7], suggesting that therapies that mitigate oxidative stress would increase NO synthesis, improve FMD, and reduce CVD risk.

We hypothesized that FMD responses would improve following a single session of LDL apheresis in hypercholesterolemic patients undergoing chronic treatment by lowering oxidative stress and improving NO bioavailability, and these benefits would persist until the subsequent apheresis session 14 d later. To test this hypothesis, hypercholesterolemic patients underwent testing of VEF and biomarkers of oxidative stress and NO metabolism prior to, immediately following, and 1, 3, 7, and 14 d following a single LDL apheresis session.

## 2. Material and Methods

### 2.1. Patients

The Institutional Review Board at Hartford Hospital approved the study. Five (4 men; 1 postmenopausal woman) of 16 active patients in the Hartford Hospital LDL apheresis program agreed to participate and provided written, informed consent. All five patients had presence of coronary artery disease, including prior coronary artery bypass graft surgery (*n* = 2), percutaneous transluminal coronary angioplasty (*n* = 1), and/or myocardial infarction (*n* = 1). Three patients had hypertension, one had prior stroke, and one had congestive heart failure. No patients currently smoked or had diabetes mellitus. Patients had undergone biweekly LDL apheresis for 3 months to 9.6 y (Table 1).

### 2.2. Study Procedures

Patients fasted for ≥8 h and were asked to refrain from taking dietary supplements or any medications that target the cardiovascular system (e.g., beta blockers, nitrates, calcium channel blockers, and angiotensin-converting-enzyme inhibitors) on the morning of each study visit. Body mass was measured to the nearest 0.1 kg on a calibrated scale and height was measured using a stadiometer. Blood pressure and heart rate were measured twice, separated by 2 min, using an automated blood pressure monitor (Welch Allyn; Skaneateles Falls, NY) after resting for 10 min in the supine position. Brachial artery FMD and plasma biomarkers were measured prior to treatment (Pre), within 1 h of completion (Post) of a single LDL apheresis session, and at 1, 3, 7, and 14 d Post. During the LDL apheresis session, blood was removed from the patient, lipid-rich plasma separated from whole blood, apolipoprotein B-containing lipoproteins cleared by dextran sulfate adsorption (DSA) (Liposorber System; Kaneka Pharma America, LLC; New York, NY), and lipid-poor plasma returned to the patient. Each LDL apheresis session lasted 3-4 h. Following measurements at Pre, patients were permitted a light snack and noncaffeinated beverages during the 4 h treatment session. Brachial artery FMD and plasma biomarkers were measured in the morning at 1, 3, 7, and 14 d Post and occurred at the same time of day (±1 h) as measurements obtained at Pre.

### 2.3. Brachial Artery FMD

Brachial artery FMD was measured following established guidelines [8] as described [9, 10]. Briefly, following a 10 min of supine rest, the right brachial artery was imaged 1–3 inches proximal to the olecranon process using a 5 to 12 MHz multifrequency linear-array transducer attached to a high-resolution ultrasound machine (Terason t3000; Burlington, MA). Resting brachial artery diameter and velocity were simultaneously measured for 1 min before rapid inflation (200 mmHg, 5 min) of a pneumatic cuff placed around the forearm immediately distal to the olecranon process. Diameter and velocity recordings resumed 1 min before cuff deflation and continued for 3 min after deflation. End-diastolic arterial diameters and velocities were analyzed using Brachial Analyzer software (Medical Imaging Applications LLC; Coralville, IA). Peak arterial diameter was calculated as the highest 3-frame average following cuff release. Brachial FMD was calculated as the absolute and percent change in diameter from resting to peak diameter. Velocity matched to the corresponding diameter was used to calculate shear rate (4*∗*velocity/diameter), an estimate of shear stress without blood viscosity. To quantify the stimulus underlying FMD, postocclusion area under the shear rate curve (SR_AUC_) was calculated, using simultaneous diameter and velocity measurements until the time that peak diameter was observed [11, 12].

### 2.4. Blood Analyses

Blood was collected by venipuncture into tubes containing sodium heparin. Tubes were centrifuged, plasma was collected, and cryovials were sent to Clinical Laboratory Partners at Hartford Hospital for analysis of lipids or stored at −80°C until analyzed.

### 2.5. Materials

HPLC-grade solvents, ascorbic acid, DTPA, methylmonoarginine,* o*-phthalaldehyde, PCA, potassium hydroxide, and potassium phosphate were purchased from Fisher Scientific (Pittsburgh, PA, USA).

### 2.6. Vitamin E and Nitro-*γ*-tocopherol

Vitamin E (as *α*- and *γ*-tocopherol) and nitro-*γ*-tocopherol were measured as described [13], with minor modifications. Plasma was extracted with hexane following saponification in the presence of ascorbic acid and then analyzed on a Prominence UPLC-MS system (Shimadzu) equipped with an autosampler maintained at 4°C (SIL-30AC), a degassing unit (DGU-20A5), a column oven set to 35°C (CTO-30A), two LC-30AD pumps, and a LCMS-2020 single quadrupole mass spectrometer. Instrument control was performed using Shimadzu LabSolution (Version 5.4). Separation was performed at 0.3 mL/min on a Kinetex C18 column (100 × 2.1 mm, 2.6 *μ*m; Phenomenex) using 60 : 40 acetonitrile : methanol as the mobile phase. Detection was performed by single ion monitoring following negative ionization with a dual ion source at the following mass-to-charge ratios (*m*/*z*): *α*-T, 429.4; *γ*-T, 415.4; nitro-*γ*-T, 460.4; and dl-tocol, 387.4 (internal standard). Nebulizing and drying gases were supplied at 1.5 and 15 L/min, respectively, and heating block and desolvation line temperatures were 500°C and 300°C.

### 2.7. Malondialdehyde

Plasma malondialdehyde (MDA), a marker of lipid peroxidation, was measured as described [13], with minor modifications using a HPLC-FL system consisting of a Waters Alliance 2695 Separations Module with a Waters 474 Scanning Fluorescence Detector (532/553 nm, excitation/emission). HPLC separation was performed at 1.0 mL/min on a Luna C18(2) column (250 × 4.6 mm, 5 *μ*m; Phenomenex) using 50 : 50 methanol and 25 mM phosphate buffer (pH 6.5) as the mobile phase.

### 2.8. Nitric Oxide Homeostasis

Arginine (ARG), the amino acid required for NO biosynthesis [14], and asymmetric dimethylarginine (ADMA), an endogenously produced competitive inhibitor of NO synthase (NOS), were simultaneously measured by HPLC as described [10], with minor modifications. In brief, ARG and ADMA were extracted from plasma (100 *μ*L) by solid-phase extraction on a polymeric cation-exchange column (HyperSep Retain-CX SPE column; 30 mg, 1 mL; Fisher Scientific) using ammonia : water : methanol (10 : 40 : 50, v : v : v). HPLC separation was performed isocratically at 1.3 mL/min on a Shimadzu LC-20ADXR system equipped with a RF-20AXL fluorescence detector programmed to 340/455 nm (excitation/emission) and a Kinetex XB-C18 column (50 × 3.0 mm, 2.6 *μ*m; Phenomenex). o-Phthalaldehyde-derivatives of ARG and ADMA were eluted using 50 mmol/L potassium phosphate buffer (pH 6.5) and 6.5% (v : v) acetonitrile as the mobile phase. After the peak of ARG eluted from the column, the gain setting was increased at 3 min to enable the detection of ADMA. After the last peak of interest eluted, the column was washed with 50% acetonitrile for 2 min and the system was equilibrated for 2 min before the next injection. Analytes were quantified on the basis of peak area relative to internal standard (methylmonoarginine).

### 2.9. Statistical Analyses

Data (means ± SD) were analyzed by SPSS Version 19.0 (SPSS Inc., Chicago, IL, USA). Time effects for FMD and plasma responses were evaluated using 1-way repeated measures ANOVA with Bonferroni correction to evaluate pairwise differences. Multiple linear regression, controlling for with-subject repeated measures, was used to calculate correlation coefficients (*R*) as described [15]. An *α*-level of *P* ≤ 0.05 was considered statistically significant.

## 3. Results

Two patients completed the final study visit at d 13 and 15 following the initial LDL apheresis session because of scheduling difficulties. Two patients were prescribed atorvastatin in combination with ezetimibe and niacin (*n* = 1) or niacin alone (*n* = 1). The remaining three patients were considered statin intolerant due to previous muscle complaints with statin use, with one of these statin intolerant patients taking ezetimibe. One patient reported taking vitamin E (400 IU) and a multivitamin daily. No prescription changes were reported during the 2 wk intervention.

**Figure 1:**
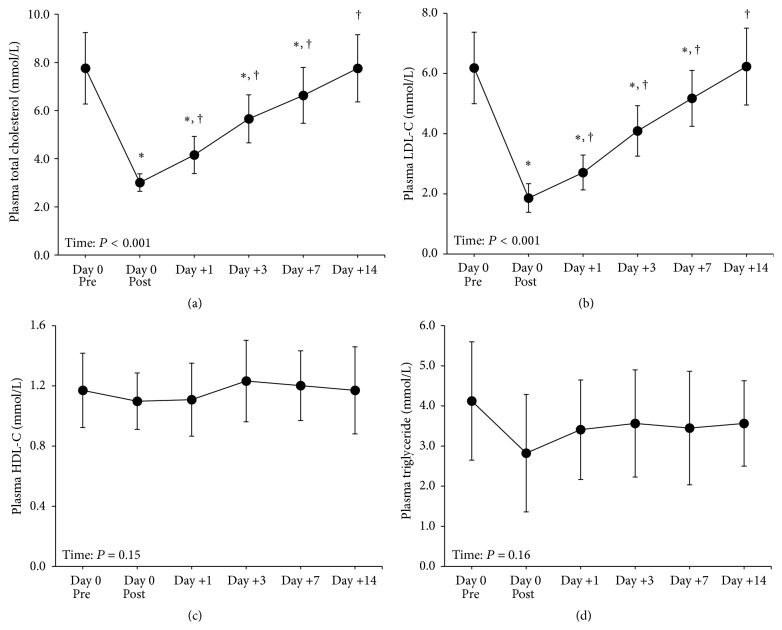
Plasma total cholesterol (a), LDL-C (b), HDL-C (c), and triglycerides (d) in patients (*n* = 5) prior to (Pre) and following LDL apheresis treatment. Data are means ± SD. LDL-C: low-density lipoprotein-cholesterol; HDL-C: high-density lipoprotein-cholesterol. ^*∗*^ *P* ≤ 0.01 from Pre; ^†^ *P* ≤ 0.01 from Post.

Relative to Pre, total C (Figure 1(a)) and LDL-C (Figure 1(b)) concentrations were lower 61% and 70%, respectively, at Post (time: *P* < 0.01). Plasma total C and LDL-C concentrations remained lower (*P* < 0.01) relative to Pre at 1, 3, and 7 d, and returned to concentrations no different from Pre by 14 d. Compared to Post, plasma total C and LDL-C concentrations were higher (*P* ≤ 0.01) at 1, 3, 7, and 14 d, demonstrating that plasma lipids rapidly increase following a single session of LDL apheresis. Plasma HDL-C concentrations (Figure 1(c)) were unaffected by treatment (*P* = 0.15). Plasma triglyceride concentrations were lower at all time points following LDL apheresis, although this was not statistically significant (*P* = 0.16) (Figure 1(d)).

**Figure 2:**
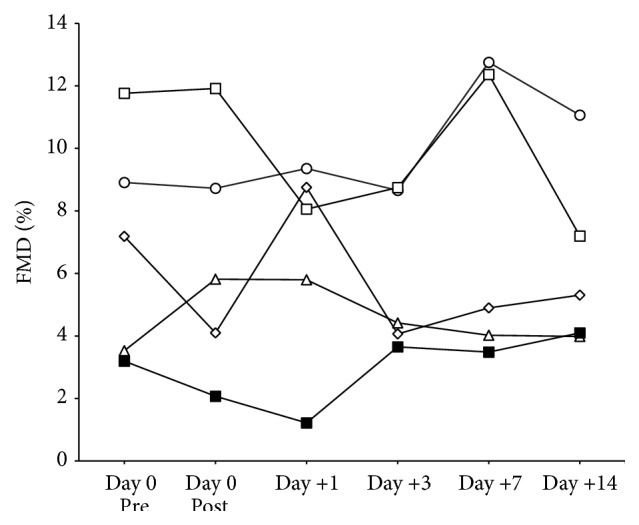
Individual changes in brachial artery flow-mediated dilation (FMD) measured prior to (Pre) and following LDL apheresis treatment.

Resting blood pressure and heart rate did not change during the study (all *P* > 0.11). Resting brachial artery diameter, peak brachial artery diameter, time to peak dilation of the brachial artery, or SR_AUC_ (i.e., the stimulus underlying FMD) did not differ from Pre at any time point following treatment (*P* = 0.13) (Table 2). FMD responses were also unaffected by LDL apheresis (time: *P* = 0.70) (Table 2) and individual responses showed little to no change in FMD immediately following treatment (Figure 2). Multiple linear regression controlling for within-subject repeated measures indicated that FMD was not related to plasma total (*R* = 0.03, *P* = 0.90) or LDL-C (*R* = 0.02, *P* = 0.93), suggesting that the reduction in circulating lipids following LDL apheresis does not immediately affect VEF.

Plasma concentrations of *γ*- and *α*-tocopherol were 52 and 63% lower (*P* < 0.01), respectively, at Post (Table 3). One day following treatment, *α*-tocopherol concentrations remained 36% lower (*P* < 0.01) whereas *γ*-tocopherol returned to levels no different than Pre (−14%; *P* > 0.05). Compared to Post, percent plasma *α*-tocopherol was higher (*P* < 0.01) at 1, 3, 7, and 14 d, whereas *γ*-tocopherol was higher (*P* < 0.01) relative to Post at 3 and 7 d.

Relative to Pre, nitro-*γ*-tocopherol decreased (*P* < 0.01) by 69% immediately following LDL apheresis treatment and remained 49% and 41% lower (*P* < 0.01) at 1 and 3 d, respectively (Table 3). Compared to Post, plasma nitro-*γ*-tocopherol was higher (*P* < 0.01) at 3, 7, and 14 d. Plasma *α*- (*R* = 0.86, *P* < 0.01) and *γ*-tocopherol (*R* = 0.63, *P* < 0.01) concentrations were directly related to plasma LDL-C, probably because tocopherol is transported in LDL particles. Furthermore, plasma nitro-*γ*-tocopherol concentrations were also directly related to plasma LDL-C (*R* = 0.77, *P* < 0.01), suggesting that acute cholesterol lowering decreased nitrative stress. Plasma tocopherols were not related to FMD (*R* = 0.07–0.14, *P* = 0.49–0.75), suggesting that reductions in antioxidants and nitrative stress following LDL apheresis do not alter VEF. Plasma MDA, ARG, and ADMA concentrations, and the ratio of ADMA : ARG, were unaffected by treatment (Table 3). Duration of LDL apheresis treatment was not related to changes in FMD or plasma biomarkers.

## 4. Discussion

The low volume of patients receiving LDL apheresis therapy (~400 in the US [16]), particularly at a single medical center, and the inconvenience of multiple follow-up visits, limited the number of participants available for the present study. Nonetheless, contrary to our hypothesis, findings of this study in patients treated chronically for hypercholesterolemia indicate that, despite substantially lowering LDL-C, VEF and plasma markers regulating vascular homeostasis are unaffected by a single session of LDL apheresis. Indeed, we show that LDL apheresis had no effect on shear-induced FMD responses or biomarkers of NO status (ARG, ADMA) or the lipid peroxidation marker MDA. Thus, while LDL apheresis is effective in lowering CVD risk [2], the mechanism by which this occurs is likely independent of NO-dependent VEF and improvements in lipid peroxidation.

Impaired brachial artery FMD, but not structural atherosclerosis, is present in children with FH compared to age- and sex-matched controls [17], suggesting that hypercholesterolemia increases CVD risk even at a young age. Chronic LDL apheresis is a treatment option for FH patients who display markedly elevated LDL-C levels despite maximally tolerated lipid-lowering medications [18]. There are currently two LDL apheresis systems approved in the US: DSA and heparin-induced extracorporeal LDL precipitation (HELP) [18]. Both systems are similarly effective in lowering LDL-C concentrations [3], but it is unclear if these two methods are representative in terms of effects on VEF. A single LDL apheresis session by DSA increased forearm blood flow responses to intra-arterial acetylcholine infusion in seven hypercholesterolemic patients, and this increase was associated with reduced plasma oxidized LDL concentrations [4], suggesting that LDL apheresis decreases oxidative stress responses that otherwise impair NO-dependent VEF. Compared with before treatment, LDL apheresis increased acetylcholine-induced, but not basal, concentrations of plasma NO metabolites by 82% [4], suggesting that LDL apheresis in the absence of a direct pharmacologic stimulus does not augment NO production in patients treated chronically for hypercholesterolemia. We measured ARG and ADMA concentrations by HPLC and calculated ADMA : ARG, an indirect index of NO biosynthesis [14]. We observed no change in ARG and ADMA concentrations following LDL apheresis by DSA, further supporting a lack of change in NO-mediated VEF.

Our observation that marked reductions in LDL-C levels by DSA do not improve shear stress-induced VEF is in agreement with prior data showing that FMD was unaffected in six hypercholesterolemic patients treated chronically with HELP [19]. In the prior study [19], FMD measured pretreatment (7.6%) and posttreatment (8.2%) was similar to that of a normocholesterolemic group (*n* = 12; ~9%), suggesting that chronic LDL apheresis therapy normalizes VEF, thus minimizing acute changes in shear stress-induced vasoactivity with each treatment. Individual patient responses in the present study revealed little to no change in FMD immediately following LDL apheresis, suggesting that statistically significant improvements in VEF would be unlikely to occur even if our sample size was increased. The variability inherent to FMD testing and lack of an appropriate control group at baseline limits our interpretation that long-term LDL apheresis treatment normalizes VEF [19]. Future studies should determine if VEF is improved in newly diagnosed patients following their first LDL apheresis treatment session and whether improvements in VEF with long-term LDL apheresis therapy, additive of chronically lowered LDL-C concentrations [20], contribute to decreased CVD risk [2].

Vitamin E functions as a chain-breaking antioxidant and is carried in plasma as part of circulating lipoproteins [21]. Serum vitamin E concentrations are higher in FH patients (47.7 ± 18.6 *μ*mol/L) receiving chronic (3 mo–9 y) LDL apheresis treatments compared to normocholesterolemic controls (23.7 ± 3.9 *μ*mol/L) [22]. Following a single session of LDL apheresis by DSA, serum concentrations of very-low-density lipoprotein (VLDL) + LDL-C and vitamin E decreased by 74% and 63%, respectively [22]. In the present study, plasma *α*- and *γ*-tocopherol concentrations decreased by 52–63% immediately following LDL apheresis, and *α*-tocopherol remained 36% lower relative to pretreatment at 1 d. Both *α*- and *γ*-tocopherol scavenge reactive oxygen species, whereas *γ*-tocopherol additionally scavenges reactive nitrogen species [23]. Nitro-*γ*-tocopherol is a nitrative stress marker resulting from *γ*-tocopherol mediated scavenging of reactive nitrogen oxides [24]. We observed that plasma NGT remained 41% lower relative to pretreatment levels at d 3. Further study is warranted to determine if *γ*-tocopherol supplementation prevents LDL apheresis-induced lowering of vitamin E and improves VEF [13].

Prior studies investigating the effect of LDL apheresis on oxidative stress have produced conflicting data, effects potentially attributable to the heterogeneity of patients studied, influence of other lipid-lowering therapies (i.e., statins) on oxidative stress [25], or the variety of biomarkers used to assess oxidative stress. Decreased plasma oxidized LDL following LDL apheresis [4] suggests a beneficial effect of LDL apheresis on oxidative stress despite acute reductions in circulating antioxidants [22]. In contrast, plasma concentrations of the oxidative stress marker 8-iso-prostaglandin-F_2*α*_ were unaffected by LDL apheresis in six FH patients [26]. Similarly, we observed no effect of a single LDL apheresis on the plasma lipid peroxidation marker MDA.

## 5. Conclusions

In conclusion, acute cholesterol reduction by LDL apheresis did not alter VEF, oxidative stress, or NO homeostasis in patients treated chronically for hypercholesterolemia. Additional study is needed to define how alterations in vitamin E status and nitrative stress following LDL apheresis potentially regulate future CVD risk.

## Figures and Tables

**Table 1:** Patient characteristics.

Treatment duration, months	54.0 ± 41.4
Age, years	52.4 ± 11.1
Height, cm	171.5 ± 9.6
Weight, kg	98.5 ± 21.7
BMI, kg/m^2^	33.1 ± 5.1
HR, bpm	71.6 ± 11.7
SBP, mmHg	129.5 ± 9.0
DBP, mmHg	79.1 ± 6.8

Data are means ± SD; *n* = 5 (4 men). BMI, body mass index; DBP, diastolic blood pressure; HR, resting heart rate; SBP, systolic blood pressure.

**Table 2:** Brachial artery responses in patients prior to (Pre) and following LDL apheresis treatment.

	Pre	Post	1 d	3 d	7 d	14 d	*P* (time)
Resting diameter, mm	4.53 ± 0.97	4.42 ± 0.96	4.46 ± 1.01	4.42 ± 1.04	4.44 ± 0.98	4.47 ± 0.98	0.13
Peak diameter, mm	4.82 ± 0.92	4.69 ± 0.92	4.74 ± 1.04	4.66 ± 1.03	4.75 ± 0.94	4.74 ± 1.00	0.18
FMD, mm	0.29 ± 0.11	0.26 ± 0.11	0.29 ± 0.15	0.24 ± 0.08	0.31 ± 0.16	0.27 ± 0.13	0.73
FMD, %	6.9 ± 3.7	6.5 ± 3.9	6.7 ± 3.3	5.9 ± 2.5	7.5 ± 4.7	6.3 ± 3.0	0.70
SR_AUC_	26556 ± 10263	22015 ± 7700	25001 ± 11530	29741 ± 10119	31183 ± 11300	25761 ± 7871	0.27

Data are means ± SD; *n* = 5. FMD, flow-mediated dilation; SR_AUC_, shear rate area under the curve.

**Table 3:** Plasma antioxidants, markers of oxidative/nitrative stress, and nitric oxide status in patients prior to (Pre) and following LDL apheresis treatment.

	Pre	Post	1 d	3 d	7 d	14 d	*P* (time)
*α*-tocopherol, *µ*mol/L	63.68 ± 46.85	21.88 ± 12.24	36.92 ± 20.58	44.50 ± 23.30^†^	50.60 ± 27.36^†^	53.77 ± 29.11^†^	<0.01
Δ*α*-tocopherol, %	—	−63.0 ± 5.1^*∗*^	−36.4 ± 14.0^*∗*†^	−23.2 ± 14.0^†^	−13.2 ± 15.2^†^	−8.6 ± 12.4^†^	<0.01
*γ*-tocopherol, *µ*mol/L	3.89 ± 2.55	1.72 ± 1.11	3.52 ± 2.87	3.75 ± 2.21	4.60 ± 2.37^†^	4.76 ± 2.48	<0.01
Δ*γ*-tocopherol, %	—	−52.2 ± 14.8^*∗*^	−14.4 ± 21.2	0.4 ± 12.3^†^	34.0 ± 51.9^†^	25.0 ± 33.3	<0.01
Nitro-*γ*-tocopherol, nmol/L	128.52 ± 53.79	39.84 ± 17.91^*∗*^	69.44 ± 43.47^*∗*^	80.39 ± 48.81^*∗*^	96.27 ± 43.29^†^	104.76 ± 41.45^†^	<0.01
Δnitro-*γ*-tocopherol, %	—	−69.2 ± 1.9^*∗*^	−49.0 ± 13.6^*∗*^	−40.8 ± 14.8^*∗*†^	−25.0 ± 13.0^†^	−16.4 ± 19.4^†^	<0.01
MDA, *µ*mol/L	1.00 ± 0.12	1.10 ± 0.17	0.94 ± 0.17	0.94 ± 0.13	1.04 ± 0.10	1.13 ± 0.20	0.17
ΔMDA, %	—	10.6 ± 15.9	−6.2 ± 10.9	−6.0 ± 15.8	4.6 ± 15.6	14.0 ± 26.1	0.18
ADMA, nmol/L	607.9 ± 171.4	610.9 ± 61.6	624.5 ± 51.7	624.2 ± 88.9	656.0 ± 116.6	571.8 ± 102.8	0.52
ΔADMA, %	—	4.2 ± 17.9	7.6 ± 23.5	5.4 ± 15.0	11.2 ± 23.6	−4.2 ± 9.6	0.26
ARG, *µ*mol/L	85.87 ± 8.21	88.36 ± 17.17	92.81 ± 7.42	88.33 ± 11.37	90.68 ± 11.85	85.12 ± 9.07	0.55
ΔARG, %	—	3.4 ± 19.7	8.4 ± 2.3	2.6 ± 7.6	5.4 ± 7.2	−0.8 ± 7.5	0.52
ADMA : ARG, nmol/*µ*mol	7.01 ± 1.33	7.12 ± 1.52	6.74 ± 0.54	7.11 ± 0.96	7.23 ± 0.84	6.74 ± 1.11	0.90
ΔADMA : ARG, %	—	1.8 ± 13.2	−0.8 ± 21.6	3.0 ± 14.7	5.6 ± 18.2	−3.4 ± 7.9	0.86

Data are means ± SD; *n* = 5. Δ, relative change from Pre; ADMA, asymmetric dimethylarginine; ARG, arginine; MDA, malondialdehyde. ^*∗*^ *P* ≤ 0.01 from Pre; ^†^ *P* ≤ 0.01 from Post.
